# Acceptability of intranasal live attenuated influenza vaccine, influenza knowledge and vaccine intent in The Gambia

**DOI:** 10.1016/j.vaccine.2018.02.037

**Published:** 2018-03-20

**Authors:** Edwin P. Armitage, Janko Camara, Sulayman Bah, Alice S. Forster, Ed Clarke, Beate Kampmann, Thushan I. de Silva

**Affiliations:** a Vaccines and Immunity Theme, Medical Research Council Unit The Gambia, PO Box 273, Banjul, Gambia; b Research Department of Behavioural Science and Health, University College London, Gower Street, London WC1E 6BT, UK; c Centre of International Child Health, Section of Paediatrics, Department of Medicine, Imperial College London, St Mary’s Campus, London W2 1PG, UK

**Keywords:** Influenza, Vaccine acceptability, Intranasal live attenuated influenza vaccine, Knowledge, attitudes and perceptions, Health Belief Model, The Gambia

## Abstract

**Background:**

The burden of influenza is increasingly recognised in Africa. The WHO recommends introducing influenza vaccination to high-risk groups: pregnant women, children <5 years, and the elderly. The Gambia currently has no influenza vaccination policy, but the NASIMMUNE study, a clinical trial of intranasal live attenuated influenza vaccines (LAIV) in young children provided an opportunity to study maternal attitudes towards LAIV for the first time in sub-Saharan Africa. We assess acceptability of LAIV, influenza knowledge and attitudes towards influenza vaccination in Gambian women. Additionally, we investigate predictors of willingness to receive influenza vaccine (intent) in pregnancy or seasonally for children <5.

**Methods:**

A cross-sectional survey was conducted in Gambian women at two urban health facilities. To assess LAIV acceptability, the exposure group (women whose children had received LAIV during the NASIMMUNE study) were compared to a control group (women whose children were not enrolled in the NASIMMUNE study). Demographics and health belief constructs were analysed as predictors of influenza knowledge and vaccine intent.

**Findings:**

The exposure group (n = 150) expressed a higher preference for a nasal spray vaccine than an injection compared to the control group (n = 304) (93.3% vs. 34.9%, OR = 26.15, p < 0.0001). Those in the exposure group who preferred the nasal spray found it less distressing, safer or equally safe, and easier or equally easy to give (all p < 0.001) than injections. Influenza knowledge increased with education level (p = 0.006 for higher education vs. none), and varied between sites (p = 0.0005). Vaccine intent was >98%, but no association with influenza knowledge or difference between groups was observed. Various health belief constructs were associated with vaccine intent.

**Conclusion:**

LAIV acceptability was higher in those with first-hand experience. Influenza vaccine intent was also high. Incorporation of seasonal LAIV into the childhood immunisation schedule in The Gambia would be feasible, particularly if combined with community-based health education.

## Highlights

• Mothers of children who received LAIV preferred intranasal vaccines over injections.
• The LAIV was viewed as safe, easy to give and less distressing than injections.
• Influenza knowledge was higher in those with more education.
• Influenza vaccine intent was very high, but not associated with influenza knowledge.
• Based on acceptability, introducing influenza vaccination would be feasible here.

## 1 Introduction

Influenza is a major cause of acute respiratory infection globally, leading to a significant burden of morbidity and mortality [1]. Seasonal influenza has been well-studied in high- and middle-income countries, but neglected in Africa [2]. The rate of influenza-associated hospitalisations in children <5 years of age is approximately 3-fold higher in low- and middle-income countries (LMIC) compared to high-income countries [3]. One meta-analysis found that 99% of deaths attributable to influenza-associated acute lower respiratory infections occurred in LMICs [4].

As a result of the increasing awareness of influenza-related disease in LMIC, in 2012 the WHO recommended that countries should consider influenza vaccination in high-risk groups including pregnant women and children <5 years [5]. The Gambia has no influenza vaccination policy and is yet to include influenza vaccination in the Expanded Programme on Immunisation (EPI) [6], [7]. The NASIMMUNE study, a systems immunology research study in children aged 24–59 months of intranasal live attenuated influenza vaccine (LAIV) is ongoing (NCT02972957). To our knowledge, this is the first interventional study in The Gambia using intranasal LAIV and offers a unique opportunity to study, for the first time, attitudes towards a new mode of vaccine delivery in this setting.

Factors that shape parental views on vaccination impact childhood vaccination rates and having insight into parental perceptions can be useful when introducing new vaccinations to a country [8]. High vaccine uptake in The Gambia through the EPI [9] has led to reductions in invasive disease secondary to *Haemophilus influenzae* type b [10] and *Streptococcus pneumoniae* [11]. Yet as more vaccines are added to the EPI, vaccine hesitancy might be a concern [12], [13], [14]. Knowledge and attitudes surrounding influenza and influenza vaccination in The Gambia are unknown. Gaining a better understanding of these factors can help guide future influenza vaccination strategies, including maternal influenza immunisation, shown to be beneficial to both mothers and infants in recent studies from South Africa and Mali [15], [16].

The Health Belief Model (HBM) provides a framework of psychosocial constructs that may be determinants of health behaviour [17]. It posits that preventative health behaviours are more likely to be exhibited when an individual perceives they are highly susceptible to the disease, that the disease is serious, that the behaviour is beneficial, there are few barriers, and are cued into action [18]. These constructs have been shown to predict uptake of influenza vaccination and other health behaviours [19], [20], [21], [22], [23], [24]. However, emotions experienced at the point of decision-making may be as important in health behaviour as a cognitive assessment of the risk, such as worry and anticipated regret, which have also been shown to be predictive of influenza vaccination [20], [25], [26]. Few studies have used the HBM to study influenza vaccine behaviour in LMICs [27], [28], [29], [30], [31].

We aimed to compare attitudes towards the safety, ease of use, and tolerability of LAIV between mothers with direct experience of their children receiving LAIV and mothers without such experience, and to establish whether their attitudes towards intranasal vaccines differ. Additionally, we aimed to quantify knowledge about influenza and to determine possible relationships between influenza knowledge, socio-demographic factors and willingness to accept influenza vaccination in pregnancy or for their children <5 (vaccine intent). Finally, we aimed to explore the relationship between health belief constructs and influenza vaccine intent in this cohort.

## 2 Methods

### 2.1 Study design

A cross-sectional survey was conducted in women ≥18 years at two sites (Sukuta and Faji Kunda health centres) five kilometres apart, in the coastal region of The Gambia in August and September 2017. In Sukuta, where NASIMMUNE was conducted, mothers of children who had been vaccinated with the intranasal LAIV (Nasovac-S®, Serum Institute of India Pvt. Ltd.) as part of the study were contacted sequentially and invited to an interview, up to a total of 150 (exposure group). A further 154 women from Sukuta who had not participated in the NASIMMUNE study were recruited at the same health centre. Due to wider community sensitization regarding the NASIMMUNE trial carried out in Sukuta, this group may have had some exposure to information about influenza and LAIV, therefore an additional control group of 150 women were recruited at Faji Kunda health centre (where there could have been no exposure to the study or community sensitization). These women were recruited through opportunistic sampling when attending for routine healthcare. The sample size was determined by the availability of mothers of children in the NASIMMUNE study (n = 168), with a 1:2 exposure to control ratio. Inclusion criteria were having at least one child <5, maternal age ≥18, resident in the area, and fluency in Mandinka (the most commonly spoken local language in the areas).

### 2.2 Data collection

Face-to-face interviews were conducted by trained field-workers who entered data in real-time into a questionnaire designed in REDCap™ [32]. The questionnaire was developed using simple terminology and was refined through cognitive pre-testing with field-workers and test participants. The questionnaire included seven sections: inclusion criteria, socio-demographics, vaccine intent, influenza knowledge, health-seeking behaviour, health beliefs, and LAIV acceptability. The influenza knowledge questions were designed *de novo* for this setting, selected and simplified from influenza knowledge questions used previously [31], [33], [34], [35] and refined following the cognitive pre-testing phase. Questions asked about symptoms, transmission, prevention, treatment, risk, and vaccination (see supplementary material: Appendix A). The responses were collated to form a score out of 15 points, which was converted to a percentage for analysis (score divided by 15, multiplied by 100).

Influenza vaccine intent was assessed for pregnancy and for children <5 by asking participants to respond to two statements: “If I was pregnant, I would get a flu vaccine if it was free” and “I would get a flu vaccine for my child under 5, every year, if it was free”. Answers were recorded on a 4-point scale where 1 = agree strongly, 2 = agree, 3 = disagree and 4 = disagree strongly, with a “don’t know” option. In follow-up questions, unprompted reasons given for answers were coded into predefined categories by the interviewer.

Health belief constructs were assessed using statements answered on the same 4-point scale. HBM constructs assessed were: perceived susceptibility, severity, benefit, barriers and cues to action. Two additional concepts were also included: worry and anticipated regret. The answers given were converted into binary “agree” or “disagree” responses for analysis.

### 2.3 Ethical considerations

Ethical approval for the study was provided by The Gambia Government/MRC Joint Ethics Committee (SCC1555). Written informed consent was obtained from all participants. It was made clear that answers were confidential and anonymised, that they could withdraw at any time or decline to answer any questions.

### 2.4 Statistical analysis

Analysis was conducted using Stata® 12.0. Descriptive statistics were used to compare proportions between groups: Pearson’s chi-squared test or two-tailed Fisher’s exact test (when one category had <5 participants) for categorical data; and Student’s *t*-test or Wilcoxon rank-sum test for normally and non-normally distributed continuous data respectively. Univariate linear regression analysis was performed for predictors of influenza knowledge. Significance-testing was used for selection of variables to include in the multivariate model at a level of p < 0.2 to minimize type II error in selection [36]. The Cochran–Mantel–Haenszel test was used to analyse variations between groups for individual influenza knowledge question responses and to evaluate the difference in preference for intranasal or injection vaccinations between groups. Two-tailed Fisher’s exact test was used to analyse perceptions of intranasal LAIV in the exposed group and associations between vaccine intent and health belief constructs. p < 0.05 was considered statistically significant.

## 3 Results

### 3.1 Participants’ characteristics

The 454 participants’ characteristics who answered the survey are displayed in Table 1. There were significant differences between the exposure and control groups with respect to age, parity, education, husband’s education and monthly household income.

**Table 1:** Socio-demographic and other characteristics of participants by group.

Participant characteristics	All n = 454 n (%)	NASIMMUNE (exposure) n = 150 n (%)	Non-NASIMMUNE (control) n = 304 n (%)	P value
Interview site:				
Sukuta	304 (67.0)	150 (100.0)	154 (50.7)	–
Faji Kunda	150 (33.0)	0 (0.0)	150 (49.3)	–
Mean% influenza knowledge score (SD)	68.0 (10.3)	69.2 (10.0)	67.4 (10.4)	0.0816*
Vaccine intent in pregnancy	447 (98.5)	150 (100.0)	297 (97.7)	0.185†
Vaccine intent for children < 5 years	448 (98.7)	146 (97.3)	302 (99.3)	0.600†

Socio-demographics:				
Mean age (SD)	28.4 (5.6)	29.5 (5.1)	27.9 (5.7)	0.0026*
Median parity (IQR)	3 (2–5)	3 (2–5)	3 (2–5)	0.0340**
Median household size (IQR)	4 (3–6)	4 (3–5)	4 (3–6)	0.0852**
Currently pregnant	36 (7.9)	15 (10.0)	21 (6.9)	0.251‡
Another pregnancy in household	111 (24.5)	33 (22.0)	78 (25.7)	0.394‡

Marital status:				
Never married	5 (1.1)	1 (0.7)	4 (1.3)	–
First (and only) wife	344 (75.8)	118 (78.7)	226 (74.3)	–
First (not only) wife	37 (8.2)	9 (6.0)	28 (9.2)	–
Second wife	58 (12.8)	18 (12.0)	40 (13.2)	–
Third or fourth wife	10 (2.2)	4 (2.7)	6 (2.0)	0.734†

Education (English school):				
None	98 (21.6)	27 (18.0)	71 (23.4)	–
Arabic school only	78 (17.2)	17 (11.3)	61 (20.1)	–
Attended primary school	50 (11.0)	17 (11.3)	33 (10.9)	–
Attended upper school	215 (47.4)	86 (57.3)	129 (42.4)	–
Higher education	13 (2.9)	3 (2.0)	10 (3.3)	0.025†

Occupation:				
None/house wife	266 (58.6)	79 (52.7)	187 (61.5)	–
Student	3 (0.7)	1 (0.7)	2 (0.7)	–
Self-employed (unskilled)	69 (15.2)	32 (21.3)	37 (12.2)	–
Self-employed (skilled)	65 (14.3)	23 (15.3)	42 (13.8)	–
Employed (salaried)	51 (11.2)	15 (10.0)	36 (11.8)	0.105†

Husband’s education:				
None	42 (9.3)	6 (4.0)	36 (11.8)	–
Arabic school only	61 (13.4)	13 (8.7)	48 (15.8)	–
Attended primary school	16 (3.5)	7 (4.7)	9 (3.0)	–
Attended upper school	259 (57.1)	89 (59.3)	170 (55.9)	–
Higher education	51 (11.2)	23 (15.3)	28 (9.2)	–
Don’t know	25 (5.5)	12 (8.0)	13 (4.3)	0.004‡

Husband’s occupation:				
None	15 (3.3)	5 (3.3)	10 (3.3)	–
Student	3 (0.7)	1 (0.7)	2 (0.7)	–
Self-employed (unskilled)	6 (1.3)	2 (1.3)	4 (1.3)	–
Self-employed (skilled)	161 (35.5)	48 (32.0)	113 (37.2)	–
Employed (salaried)	253 (55.7)	93 (62.0)	160 (52.6)	–
Don’t know	16 (3.5)	1 (0.7)	15 (4.9)	0.120†

Household income (GMD per month):				
GMD 500-GMD 4,999	71 (15.6)	20 (13.3)	51 (16.8)	–
GMD 5,000-GMD 9,999	186 (41.0)	70 (46.7)	116 (38.2)	–
GMD >10,000	100 (22.0)	44 (29.3)	56 (18.4)	–
Don’t know/unwilling to say	97 (21.4)	16 (10.7)	81 (26.6)	<0.001‡

Monthly household income stated in Gambian Dalasis (GMD). 1 USD = 48 GMD at time of writing.

* Two-group mean-comparison *t*-test (normally distributed).

** Wilcoxon rank-sum test (non-normally distributed).

† Two-tailed Fisher’s exact test (when n < 5 in some cases).

‡ Pearson's chi-squared test.

### 3.2 LAIV acceptability

**Fig. 1:**
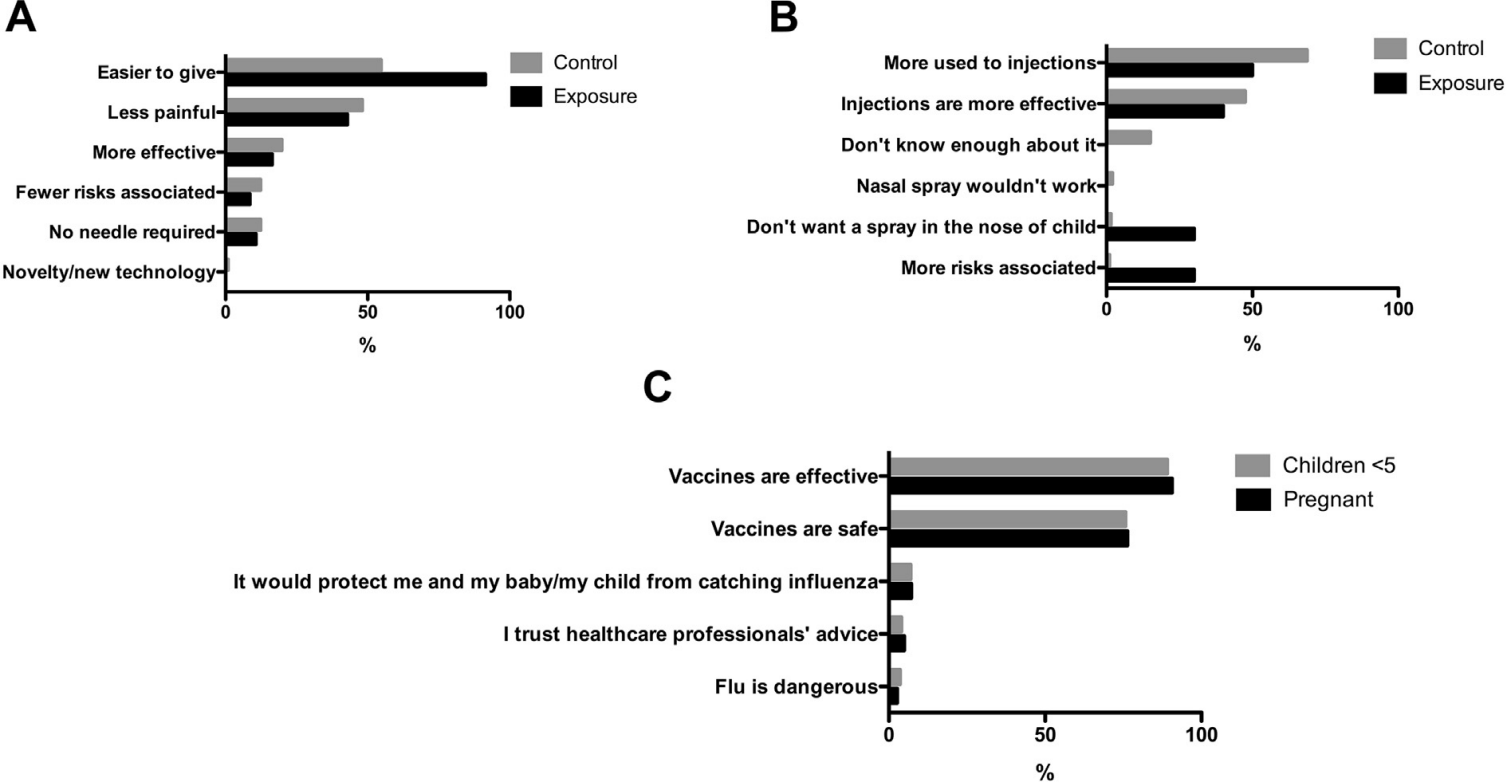
Respondents’ unprompted reasons for answers given. (A) Respondents’ unprompted reasons for preferring nasal spray by group (exposure n = 140, control n = 106). (B) Respondents’ unprompted reasons for preferring injections by group (exposure n = 10, control n = 198). (C) Respondents’ unprompted reasons for accepting an influenza vaccine during pregnancy or for their children < 5 years (pregnant n = 447, children < 5 n = 448).

When asked “If given a choice for your child between a flu vaccine injection and a nasal spray, which would you prefer?” a significantly higher proportion of the exposure group stated they would prefer a nasal spray compared to the control group (93.3% vs. 34.9%, OR = 26.15, p < 0.0001, Table 2). The most common unprompted reasons for preferring the nasal spray were that it is easier to give, less painful and a perception of greater effectiveness (Fig. 1A). The most commonly stated reasons for preferring injections were a greater familiarity with injections and a belief that injections are more effective (Fig. 1B). In the exposure group, stating a preference for the nasal spray was associated with finding the LAIV less distressing (p < 0.001), safer or equally safe (p < 0.001) and easier or equally easy to give (p < 0.001) when compared to injections (Table 2).

**Table 2:** Intranasal LAIV preference and acceptability.

Question	Levels	NASIMMUNE (exposure) n = 150 n (%)		Non-NASIMMUNE (control) n = 304 n (%)	Cochran–Mantel–Haenszel odds ratio (95% CI, p value)
Vaccine delivery preference	Injection	10 (6.7)		198 (65.1)	ref
	Nasal spray	140 (93.3)		106 (34.9)	26.15 (11.51, 59.41, p < 0.0001)*

	Levels	Preferred injections n = 10	Preferred nasal spray n = 140		Two-tailed Fisher’s exact test p value

Less distressing	No	7 (70.0)	3 (2.1)	–	ref
	Yes	3 (30.0)	137 (97.9)	–	<0.001*
Safer or equally safe	No	8 (80.0)	2 (1.4)	–	ref
	Yes	2 (20.0)	138 (98.6)	–	<0.001*
Easier or equally easy to give	No	8 (80.0)	1 (0.7)	–	ref
	Yes	2 (20.0)	139 (99.3)	–	<0.001*

* Significant p < 0.05. ref = reference category used for baseline comparison.

### 3.3 Influenza knowledge

**Fig. 2:**
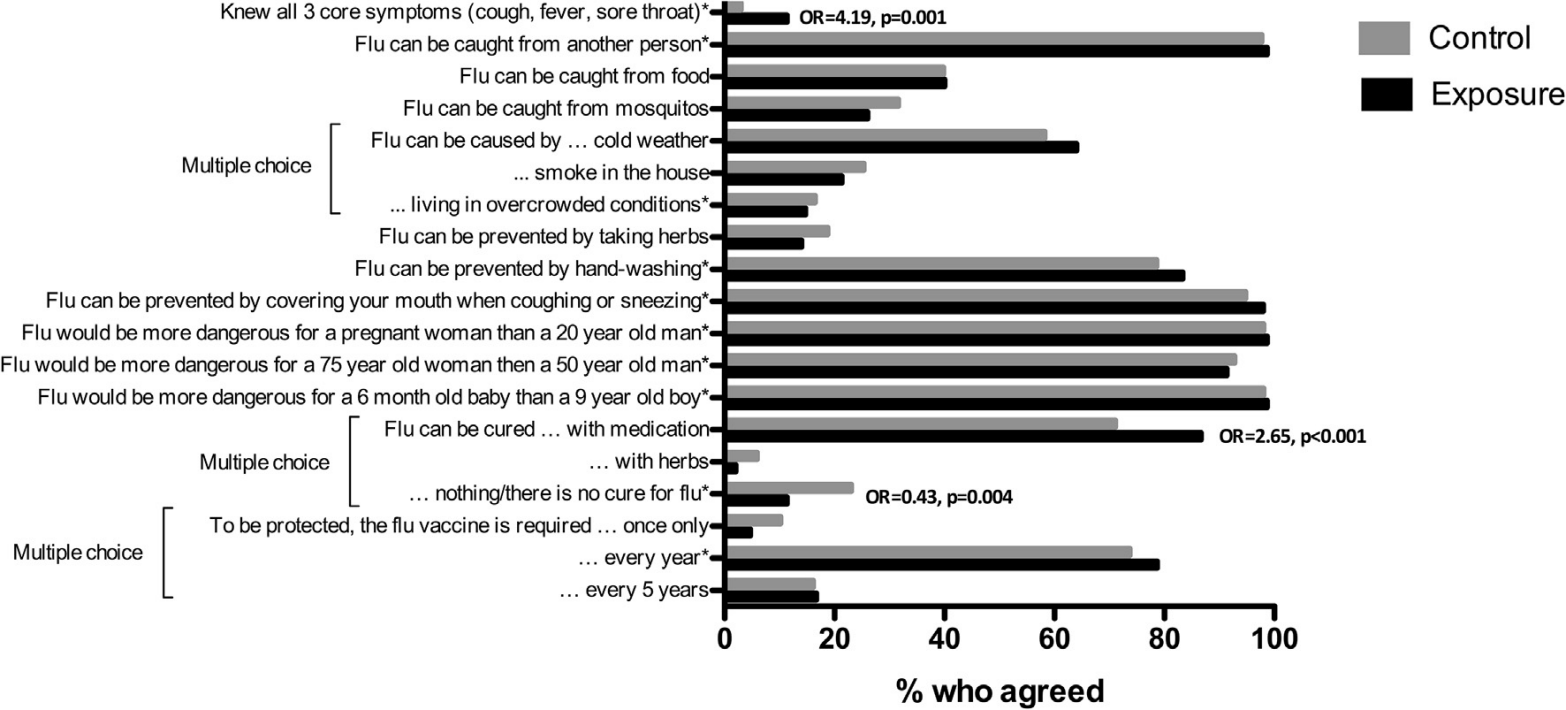
Individual influenza knowledge question responses by group. Cochran–Mantel–Haenszel odds ratios and p values shown only for questions where groups differed significantly. ^*^Correct statements.

The mean percentage influenza knowledge score was 68.0% and was not significantly different between exposure and control groups (69.2% vs. 67.4%, p = 0.0816), although differences were seen in individual questions (Fig. 2). Participants recruited at Faji Kunda had significantly higher scores than at Sukuta (70.4% vs. 66.8%, p = 0.0005). When comparing exposure and control participants from Sukuta, the knowledge was significantly higher in the exposure group (69.2% vs. 64.4%, p = 0.0001). Of the control participants from Sukuta, only two (1.3%) had been asked to participate in NASIMMUNE but did not.

The responses to individual knowledge questions are shown in Fig. 2. A significantly higher proportion of exposure participants could name all three core symptoms of influenza (11.3% vs. 3.0%, OR = 4.19, p = 0.001), while a significantly lower proportion of exposure group participants correctly identified that there is no cure for influenza (11.3% vs. 23.0%, OR = 0.43, p = 0.004) but thought “medication” could cure influenza (86.7% vs. 71.1%, OR = 2.65, p < 0.0001). There were no significant differences between groups in any other questions.

Results of the univariate and multivariate linear regression analysis of factors that predict influenza knowledge are presented in Table 3. In the multivariate linear regression model, there was a significantly higher level of influenza knowledge in participants who had attended Arabic school, upper school or had higher education compared to no education, exhibiting an increased score with each stepwise increase in educational level. Additionally, participants whose husbands were students showed a significantly higher knowledge score than those whose husbands had no occupation (β = 13.31, p = 0.032). The multivariate model explained a significant proportion of the variance in influenza knowledge (adjusted R^2^ = 0.1165, p < 0.0001).

**Table 3:** Predictors of influenza knowledge.

Predictors			Linear regression					
Total n = 453 (n = 1 excluded due to missing data in knowledge questions)		Influenza knowledge score	Univariate analysis			Multivariate analysis†		
Continuous/discrete		mean %	β	95% CI	p value	β‡	95% CI‡	p value‡
Age (in years)		–	0.02	−0.15, 0.19	0.788	–	–	–
Parity		–	−0.67	−1.13, −0.19	0.006*	−0.16	−0.66, 0.35	0.546
Number of people living in household		–	−0.25	−0.66, 0.16	0.236	–	–	–

Categorical	Levels							

Group and site	Sukuta (exposure)	69.2	ref	–	–	ref	–	–
	Sukuta (control)	64.4	−4.71	−6.96, −2.46	<0.001*	−4.36	−6.65, −2.07	<0.001**
	Faji Kunda (control)	70.4	1.20	−1.06, 3.46	0.298	1.49	−0.90, 3.88	0.220

Marital status	Never married	68.0	ref	–	–	–	–	–
	First and only wife	68.0	−0.01	−9.13, 9.11	0.998	–	–	–
	First wife (not only)	67.9	−0.07	−9.72, 9.57	0.988	–	–	–
	Second wife	67.2	−0.76	−10.19, 8.68	0.875	–	–	–
	Third or fourth wife	71.3	3.33	−7.76, 14.42	0.555	–	–	–

Educational level	None	63.5	ref	–	–	ref	–	–
	Arabic school only	67.4	3.88	0.93, 6.83	0.010*	4.32	1.23, 7.42	0.006**
	Attended primary school	67.3	3.88	0.48, 7.28	0.026*	3.40	−0.03, 6.83	0.052
	Attended upper school	69.8	6.36	3.99, 8.73	<0.001*	6.08	3.49, 8.68	<0.001**
	Higher education	76.9	13.45	7.71, 19.19	<0.001*	9.71	2.84, 16.59	0.006**

Occupation	None	67.9	ref	–	–	ref	–	–
	Student	80.0	12.13	0.46, 23.79	0.042*	5.40	−6.53, 17.32	0.374
	Self-employed (unskilled)	66.8	−1.11	−3.83, 1.60	0.422	−1.06	−3.76, 1.64	0.443
	Self-employed (skilled trade)	67.2	−0.69	−3.48, 2.09	0.624	0.04	−2.69, 2.77	0.977
	Employed (salaried)	70.3	2.45	−0.62, 5.52	0.117*	0.05	−3.25, 3.35	0.977

Husband’s educational level	None	67.1	ref	–	–	ref	–	–
	Arabic school only	65.9	−1.24	−5.27, 2.78	0.545	−2.62	−6.59, 1.35	0.195
	Attended primary school	66.2	−0.89	−6.79, 5.00	0.766	−0.37	−6.02, 5.28	0.898
	Attended upper school	68.2	1.05	−2.29, 4.39	0.538	−0.65	−3.98, 2.69	0.704
	Higher education	71.4	4.23	0.05, 8.41	0.048*	0.61	−3.73, 4.95	0.783
	Don’t know	66.1	−1.01	−6.08, 4.06	0.696	−3.23	−8.44, 1.98	0.223

Husband’s occupation	None	65.8	ref	–	–	ref	–	–
	Student	77.8	12.00	−0.78, 24.78	0.066*	13.31	1.17, 25.46	0.032**
	Self-employed (unskilled)	70.0	4.22	−5.54, 13.99	0.396	5.19	−4.32, 14.70	0.284
	Self-employed (skilled trade)	67.7	1.88	−3.57, 7.34	0.498	3.83	−1.39, 9.05	0.150
	Employed (salaried)	68.1	2.29	−3.08, 7.66	0.403	3.23	−1.92, 8.37	0.218
	Don’t know†	68.9	3.11	−4.27, 10.49	0.408	5.31	−2.08, 12.70	0.159

Household income ( GMD per month)	GMD 500-GMD 4,999	66.5	ref	–	–	ref	–	–
	GMD 5,000-GMD 9,999	68.2	1.59	−1.23, 4.40	0.269	1.09	−1.64, 3.82	0.432
	GMD >10,000	69.2	2.68	−0.46, 5.82	0.094*	1.72	−1.36, 4.79	0.272
	Don’t know/unwilling to say	67.6	1.15	−2.00, 4.30	0.474	1.10	−1.98, 4.18	0.481

Vaccination intent	Levels							

“I would get a flu vaccine in pregnancy if available for free”	Disagree	66.7	ref	–	–	–	–	–
	Agree	67.9	1.28	−7.80, 10.36	0.782	–	–	–
“I would get a flu vaccine for my child under 5, every year, if it was free”	Disagree	66.7	ref	–	–	–	–	–
	Agree	68.0	1.37	−10.27, 13.00	0.817	–	–	–

† Predictors included in the multivariate linear regression model (p values < 0.2): parity, group/site, educational level, occupation, husband’s educational level, husband’s occupation and monthly household income.

‡ Adjusted values.

* p < 0.2.

** Significant p < 0.05. ref = reference category used for baseline comparison.

### 3.4 Influenza vaccination intent

Almost all respondents stated that they agreed or agreed strongly with the vaccine intent statements (98.5% and 98.7% for pregnancy and children <5 respectively) and there was no difference in vaccine intent between groups (Table 1). Unprompted reasons given for intent are shown in Fig. 1C. There was no association between influenza knowledge and vaccine intent for pregnancy or children <5 as shown in Table 3 (p = 0.782 and p = 0.817 respectively).

The associations between health belief concepts and vaccine intent are presented in Table 4. For children <5, significant associations were seen for perceived susceptibility, severity, benefit, and barriers, while for pregnancy, only perceived susceptibility, severity and worry were significantly associated with vaccine intent.

**Table 4:** Health belief constructs as predictors of vaccine intent.

Health belief construct	Question wording	Levels	Vaccine intent†		Two-tailed Fisher’s exact p value
			Disagree n (%)	Agree n (%)	
*Children < 5*					
Perceived susceptibility	“If my child under 5 had not been given the flu vaccine, I would expect them to get flu”	Disagree	4 (100.0)	30 (6.7)	<0.001*
		Agree	0 (0.0)	415 (93.3)	

Perceived severity	“If my child under 5 caught flu, it would be more mild than in the general public”	Disagree	2 (50.0)	188 (42.4)	1.000
		Agree	2 (50.0)	257 (57.8)	
	“If my child under 5 caught flu, they might need to be admitted to hospital”	Disagree	1 (25.0)	4 (0.9)	0.044*
		Agree	3 (75.0)	441 (99.1)	

Perceived benefit	“If my child under 5 was given the flu vaccine, it would prevent them catching flu”	Disagree	3 (75.0)	3 (0.7)	<0.001*
		Agree	1 (25.0)	441 (99.3)	

Perceived barriers	“If my child under 5 had been given the flu vaccine, the vaccine could give them flu”	Disagree	2 (50.0)	404 (91.0)	0.046*
		Agree	2 (50.0)	40 (9.0)	
	“The flu vaccine is unsafe for children”	Disagree	2 (50.0)	406 (92.1)	0.036*
		Agree	2 (50.0)	35 (7.9)	

Cues to action	“If a nurse or doctor recommended the flu vaccine during pregnancy or for my child under 5, I would agree to get it because of what they said”	Disagree	1 (25.0)	202 (45.1)	0.631
		Agree	3 (75.0)	246 (54.9)	
	“If my friends or relatives recommended the flu vaccine during pregnancy or for my child under 5, I would get it because of what they said”	Disagree	3 (100.0)	336 (75.3)	1.000
		Agree	0 (0.0)	110 (24.7)	

Worry	“If my child under 5 hadn't been given the flu vaccine, I would worry about them getting flu”	Disagree	1 (25.0)	13 (2.9)	0.119
		Agree	3 (75.0)	434 (97.1)	

Anticipated regret	“If I refused to get the flu vaccine for my child under 5, but then they got sick with flu, I would be angry with myself”	Disagree	1 (25.0)	14 (3.1)	0.127
		Agree	3 (75.0)	433 (96.9)	

*Pregnancy*					
Perceived susceptibility	“If I was pregnant but hadn't been given the flu vaccine, I would expect to get flu”	Disagree	3 (50.0)	30 (6.9)	0.006*
		Agree	3 (50.0)	407 (93.1)	

Perceived severity	“If I was pregnant and caught flu, it would be more mild than in the general public”	Disagree	0 (0.0)	200 (45.1)	0.036*
		Agree	6 (100.0)	244 (55.0)	

Perceived benefit	“If I had been given a flu vaccine during pregnancy, it would prevent me catching flu”	Disagree	0 (0.0)	5 (1.1)	1.000
		Agree	6 (100.0)	440 (98.9)	
	“If I got a flu vaccine during pregnancy, it would protect my baby from getting flu in the first few months of life”	Disagree	0 (0.0)	5 (1.13)	1.000
		Agree	6 (100.0)	439 (98.9)	

Perceived barriers	“The flu vaccine is unsafe during pregnancy”	Disagree	4 (66.7)	402 (91.6)	0.090
		Agree	2 (33.3)	37 (8.4)	
	“If I got a flu vaccine during pregnancy, the vaccine could give me flu”	Disagree	5 (83.3)	414 (93.7)	0.332
		Agree	1 (16.7)	28 (6.3)	

Cues to action	“If a nurse or doctor recommended the flu vaccine during pregnancy or for my child under 5, I would agree to get it because of what they said”	Disagree	1 (16.7)	203 (45.4)	0.230
		Agree	5 (83.3)	244 (54.6)	
	“If my friends or relatives recommended the flu vaccine during pregnancy or for my child under 5, I would get it because of what they said”	Disagree	4 (66.7)	337 (75.9)	0.635
		Agree	2 (33.3)	107 (24.1)	

Worry	“If I was pregnant and hadn't been given the flu vaccine, I would worry about getting flu”	Disagree	3 (50.0)	18 (4.0)	0.002*
		Agree	3 (50.0)	429 (96.0)	

Anticipated regret	“If I was pregnant and refused to get the flu vaccine, but then got sick with flu, I would be angry with myself”	Disagree	1 (16.7)	24 (5.4)	0.290
		Agree	5 (83.3)	423 (94.6)	

† Participants who answered “don’t know” to health belief construct or vaccine intent questions omitted.

* Significant p < 0.05.

## 4 Discussion

There are few countries with seasonal influenza vaccination in sub-Saharan Africa, with none currently using intranasal LAIV [37]. The NASIMMUNE study, ongoing in The Gambia, provided an opportunity to study perceptions of intranasal LAIV between mothers whose children had recently received LAIV and women with no experience of LAIV. In the latter group, preference for an intranasal vaccine was moderate at 34.9%, with the most commonly stated reasons for preferring an injection relating to familiarity with injections and beliefs of greater effectiveness. Our results show that in those with direct experience of LAIV, the preference for nasal spray was significantly higher, in keeping with studies in high income countries [38], [39]. Women in the exposure group stated, unprompted, that nasal sprays were easier to give than injections and that they were less painful, suggesting that the LAIV given in NASIMMUNE was viewed favourably. These results indicate that a future influenza vaccination programme in The Gambia using intranasal LAIV in children <5 would be received positively, particularly if the introduction were coupled with demonstrations or educational sessions to promote their safety, ease of use and effectiveness.

The influenza knowledge questions covered a range of influenza-related topics including symptoms, transmission, severity, high-risk groups, treatment and vaccination. The educational level in The Gambia is generally low, with many women illiterate, and never attending school (21.6%). The mean knowledge scores were no different between the exposure and control groups, but a good understanding of disease and health-risk concepts was seen overall despite the low formal education level.

Due to community sensitization and possible knowledge transfer between NASIMMUNE study participants and non-study participants in Sukuta, a control group from Faji Kunda who had had no exposure to any trial information about influenza were included. Surprisingly, Faji Kunda had significantly higher knowledge than Sukuta overall. The effect was independent of education, which was broadly similar between sites, although knowledge did increase significantly with more education at both sites. The reason for the higher scores in Faji Kunda is unclear, but health beliefs within in the two communities may differ significantly and previous studies conducted at Faji Kunda may have influenced the results. Several large vaccine trials have recently recruited children through the Faji Kunda EPI clinic, which have included health, hygiene and nutritional education as part of community sensitization. One study from The Gambia has shown that such activities effectively disseminate information throughout the community [40], while others have shown that community social networks are important to knowledge, particularly when access to external information is limited [41]. Furthermore, educational women’s groups can significantly improve health outcomes in low-income settings [42], [43]. Community health beliefs may, therefore, be as important as formal education in regard to influenza-related knowledge.

When comparing groups from Sukuta alone, influenza knowledge was significantly higher in the exposure group, suggesting that participation in the NASIMMUNE study may have increased knowledge about influenza and vaccination. However, when asked what can cure influenza, a significantly higher proportion of the exposure group incorrectly answered “medication”, rather than “nothing/no treatment”. This could indicate that involvement in the study led people to erroneously believe that specific anti-influenza medications are available. Children presenting with cough, fever and rhinorrhoea in the follow-up period of the NASIMMUNE study were commonly given paracetamol, which may have been mistakenly regarded as a “cure”, possibly explaining the finding. This is a reminder that clinical trials can inadvertently spread misinformation in these settings and that steps should be taken to mitigate this risk through careful provision of information during informed consent processes and throughout studies [44], [45], [46], [47].

Influenza vaccine intent was assessed by asking participants if they would accept an influenza vaccine if it was freely available for themselves during pregnancy or for their children <5. Over 98% of participants responded that they would accept the vaccination. The high intent could be explained by the clinical trials that have been conducted at both health centres for many years, which may have led to a high level of trust in healthcare in these communities. To avoid response bias, participants were reassured during the interview that they should not feel pressured to answer in the affirmative. Nonetheless, the high influenza vaccine intent observed may be, in part, due to acquiescence bias or social desirability bias. However, the unprompted stated reasons for their answers confirmed that there is a strong belief that vaccines are safe and effective, and related to vaccines generally rather than influenza vaccines specifically. The high intent is also consistent with the high EPI coverage in The Gambia (95% for BCG and 81–99% for three Diphtheria-Pertussis-Tetanus doses) which is among the highest in Africa [9]. Neither higher influenza knowledge nor higher educational level were associated with vaccine intent. This may be due to the small numbers disagreeing with the intent statements, which also meant that the associations between health belief constructs and vaccine intent could not be quantified, but our results confirm previous findings elsewhere [19], [20]. A larger sample size would be required to determine which health belief constructs are most predictive of vaccine intent in this setting.

A key limitation to this study is that opportunistic sampling was used. The sample may therefore not be representative of the wider community, which could explain the small observed differences in socio-demographic makeup between groups. The higher educational level and income observed in the exposure group may be due to more educated and affluent people being more willing to participate in clinical trials. Future studies should use probability sampling at more sites to better represent the knowledge, attitudes and perceptions towards influenza and vaccination in the country.

Due to the high vaccine intent seen, the study was also under-powered to explore associations between intent and knowledge, so these should be interpreted cautiously. The intent questions specified that the vaccine was offered for free, so vaccine intent if the vaccine was only available for a fee remains unknown. Additionally, prior to being asked about LAIV preference over injections, they were not informed about the effectiveness of each vaccine, which may have altered their answers. Future studies could address these limitations in similar surveys, or use qualitative research methods to explore these attitudes in more depth, to gain an understanding of the underlying beliefs and motivations behind vaccine behaviour in The Gambia.

## 5 Conclusion

Willingness to undertake influenza vaccination during pregnancy, or to get annual seasonal vaccination for children <5 years if freely available was high, as was acceptability of LAIV in those with first-hand experience. Incorporation of the intranasal influenza vaccine into the childhood immunisation schedule in The Gambia in the future would likely be feasible from an acceptability perspective. Knowledge and understanding of health-related concepts surrounding influenza was generally good, though varied between communities, and was significantly related to higher educational levels. Despite reasonable health knowledge in this low-income, low-education setting, more formal education would have a positive impact on influenza and health knowledge, and potentially have wider community benefits as well. Community-based educational interventions may also be beneficial in The Gambia.

## References

[1] WHO. Fact Sheet no 211. Influenza (seasonal) Geneva: World Health Organization; 2016. Available online: <http://www.who.int/mediacentre/factsheets/fs211/en/> [accessed on 16th Oct 2017].

[2] Katz M.A., Schoub B.D., Heraud J.M., Breiman R.F., Njenga M.K., Widdowson M.A. (2012). Influenza in Africa: uncovering the epidemiology of a long-overlooked disease. J Infect Dis.

[3] Lafond K.E., Nair H., Rasooly M.H., Valente F., Booy R., Rahman M. (2016). Global role and burden of influenza in pediatric respiratory hospitalizations, 1982–2012: a systematic analysis. PLoS Med.

[4] Nair H., Brooks W.A., Katz M., Roca A., Berkley J.A., Madhi S.A. (2011). Global burden of respiratory infections due to seasonal influenza in young children: a systematic review and meta-analysis. Lancet.

[5] Meeting of the Strategic Advisory Group of Experts on immunization, April 2012 – conclusions and recommendations. Wkly Epidemiol Rec 2012;87:201–16. 24340402

[6] Schoub B.D., Gessner B.D., Ampofo W., Cohen A.L., Steffen C.A. (2013). Afriflu2 – second international workshop on influenza vaccination in the African continent – 8 November 2012, Cape Town (South Africa). Vaccine.

[7] Duque J., McMorrow M.L., Cohen A.L. (2014). Influenza vaccines and influenza antiviral drugs in Africa: are they available and do guidelines for their use exist?. BMC Public Health.

[8] Lasher H. (2001). Advocacy for immunization.

[9] Scott S., Odutola A., Mackenzie G., Fulford T., Afolabi M.O., Lowe Jallow Y. (2014). Coverage and timing of children's vaccination: an evaluation of the expanded programme on immunisation in The Gambia. PLoS One.

[10] Adegbola R.A., Secka O., Lahai G., Lloyd-Evans N., Njie A., Usen S. (2005). Elimination of Haemophilus influenzae type b (Hib) disease from The Gambia after the introduction of routine immunisation with a Hib conjugate vaccine: a prospective study. Lancet.

[11] Mackenzie G.A., Hill P.C., Sahito S.M., Jeffries D.J., Hossain I., Bottomley C. (2017). Impact of the introduction of pneumococcal conjugate vaccination on pneumonia in The Gambia: population-based surveillance and case-control studies. Lancet Infect Dis.

[12] Meeting of the Strategic Advisory Group of Experts on immunization, October 2014 – conclusions and recommendations. Wkly Epidemiol Rec 2014;89:561–76. 25513671

[13] Larson H.J., Jarrett C., Schulz W.S., Chaudhuri M., Zhou Y., Dube E. (2015). Measuring vaccine hesitancy: the development of a survey tool. Vaccine.

[14] Schmid P., Rauber D., Betsch C., Lidolt G., Denker M.L. (2017). Barriers of influenza vaccination intention and behavior – a systematic review of influenza vaccine hesitancy, 2005–2016. PLoS One.

[15] Madhi S.A., Cutland C.L., Kuwanda L., Weinberg A., Hugo A., Jones S. (2014). Influenza vaccination of pregnant women and protection of their infants. N Engl J Med.

[16] Tapia M.D., Sow S.O., Tamboura B., Teguete I., Pasetti M.F., Kodio M. (2016). Maternal immunisation with trivalent inactivated influenza vaccine for prevention of influenza in infants in Mali: a prospective, active-controlled, observer-blind, randomised phase 4 trial. Lancet Infect Dis.

[17] Becker M.H. (1974). The health belief model and personal health behavior.

[18] Rosenstock I.M., Strecher V.J., Becker M.H. (1988). Social learning theory and the Health Belief Model. Health Educ Quart.

[19] Chen M.F., Wang R.H., Schneider J.K., Tsai C.T., Jiang D.D., Hung M.N. (2011). Using the Health Belief Model to understand caregiver factors influencing childhood influenza vaccinations. J Community Health Nurs.

[20] Weinstein N.D., Kwitel A., McCaul K.D., Magnan R.E., Gerrard M., Gibbons F.X. (2007). Risk perceptions: assessment and relationship to influenza vaccination. Health Psychol.

[21] Christy S.M., Winger J.G., Raffanello E.W., Halpern L.F., Danoff-Burg S., Mosher C.E. (2016). The role of anticipated regret and health beliefs in HPV vaccination intentions among young adults. J Behav Med.

[22] Gerend M.A., Shepherd J.E. (2012). Predicting human papillomavirus vaccine uptake in young adult women: comparing the health belief model and theory of planned behavior. Ann Behav Med.

[23] Bowyer H.L., Forster A.S., Marlow L.A., Waller J. (2014). Predicting human papillomavirus vaccination behaviour among adolescent girls in England: results from a prospective survey. J Fam Plann Reprod Health Care.

[24] Livi S., Zeri F., Baroni R. (2017). Health beliefs affect the correct replacement of daily disposable contact lenses: predicting compliance with the Health Belief Model and the Theory of Planned Behaviour. Cont Lens Anterior Eye.

[25] Tucker Edmonds B.M., Coleman J., Armstrong K., Shea J.A. (2011). Risk perceptions, worry, or distrust: what drives pregnant women's decisions to accept the H1N1 vaccine?. Matern Child Health J.

[26] Chapman G.B., Coups E.J. (2006). Emotions and preventive health behavior: worry, regret, and influenza vaccination. Health Psychol.

[27] Kwong E.W., Pang S.M., Choi P.P., Wong T.K. (2010). Influenza vaccine preference and uptake among older people in nine countries. J Adv Nurs.

[28] Tuohetamu S., Pang M., Nuer X., Mahemuti Mohemaiti P., Qin Y. (2017). The knowledge, attitudes and practices on influenza among medical college students in Northwest China. Hum Vaccin Immunother.

[29] Ditsungnoen D., Greenbaum A., Praphasiri P., Dawood F.S., Thompson M.G., Yoocharoen P. (2016). Knowledge, attitudes and beliefs related to seasonal influenza vaccine among pregnant women in Thailand. Vaccine.

[30] Wong K.K., Cohen A.L., Norris S.A., Martinson N.A., von Mollendorf C., Tempia S. (2016). Knowledge, attitudes, and practices about influenza illness and vaccination: a cross-sectional survey in two South African communities. Influenza Other Respir Viruses.

[31] James P.B., Rehman I.U., Bah A.J., Lahai M., Cole C.P., Khan T.M. (2017). An assessment of healthcare professionals' knowledge about and attitude towards influenza vaccination in Freetown Sierra Leone: a cross-sectional study. BMC Public Health.

[32] Harris P.A., Taylor R., Thielke R., Payne J., Gonzalez N., Conde J.G. (2009). Research electronic data capture (REDCap) – a metadata-driven methodology and workflow process for providing translational research informatics support. J Biomed Inform.

[33] Khun M., Heng C., Md H.O., Kasuya H., Sakamoto J. (2012). Knowledge, attitudes and practices towards avian influenza A (H5N1) among Cambodian women: a cross-sectional study. Asian Pac J Trop Med.

[34] Oria P.A., Arunga G., Lebo E., Wong J.M., Emukule G., Muthoka P. (2013). Assessing parents' knowledge and attitudes towards seasonal influenza vaccination of children before and after a seasonal influenza vaccination effectiveness study in low-income urban and rural Kenya, 2010–2011. BMC Public Health.

[35] Yudin M.H., Salaripour M., Sgro M.D. (2009). Pregnant women's knowledge of influenza and the use and safety of the influenza vaccine during pregnancy. J Obstet Gynaecol Can.

[36] Maldonado G., Greenland S. (1993). Simulation study of confounder-selection strategies. Am J Epidemiol.

[37] Ortiz J.R., Perut M., Dumolard L., Wijesinghe P.R., Jorgensen P., Ropero A.M. (2016). A global review of national influenza immunization policies: analysis of the 2014 WHO/UNICEF Joint Reporting Form on immunization. Vaccine.

[38] Flood E.M., Ryan K.J., Rousculp M.D., Beusterien K.M., Block S.L., Hall M.C. (2011). A survey of children's preferences for influenza vaccine attributes. Vaccine.

[39] Dube E., Gagnon D., Kiely M., Boulianne N., Landry M. (2015). Acceptability of live attenuated influenza vaccine by vaccine providers in Quebec. Canada. Hum Vaccin Immunother.

[40] Dierickx S., O'Neill S., Gryseels C., Immaculate Anyango E., Bannister-Tyrrell M., Okebe J. (2017). Community sensitization and decision-making for trial participation: a mixed-methods study from The Gambia. Dev World Bioeth.

[41] Ackerson K., Zielinski R. (2017). Factors influencing use of family planning in women living in crisis affected areas of Sub-Saharan Africa: a review of the literature. Midwifery.

[42] Nair N., Tripathy P., Sachdev H.S., Pradhan H., Bhattacharyya S., Gope R. (2017). Effect of participatory women's groups and counselling through home visits on children's linear growth in rural eastern India (CARING trial): a cluster-randomised controlled trial. Lancet Glob Health.

[43] Tripathy P., Nair N., Sinha R., Rath S., Gope R.K., Rath S. (2016). Effect of participatory women's groups facilitated by Accredited Social Health Activists on birth outcomes in rural eastern India: a cluster-randomised controlled trial. Lancet Glob Health.

[44] Carvalho A.A., Costa L.R. (2013). Mothers' perceptions of their child's enrollment in a randomized clinical trial: poor understanding, vulnerability and contradictory feelings. BMC Med Ethics.

[45] Idoko O.T., Diallo A., Sow S.O., Hodgson A., Akinsola A., Diarra B. (2015). Community perspectives associated with the African PsA-TT (MenAfriVac) vaccine trials. Clin Infect Dis.

[46] Mboizi R.B., Afolabi M.O., Okoye M., Kampmann B., Roca A., Idoko O.T. (2017). Recall and decay of consent information among parents of infants participating in a randomized controlled clinical trial using an audio-visual tool in The Gambia. Hum Vaccin Immunother.

[47] Moodley K., Pather M., Myer L. (2005). Informed consent and participant perceptions of influenza vaccine trials in South Africa. J Med Ethics.

