# Supplementary material for: Acceptability of intranasal live attenuated influenza vaccine, influenza knowledge and vaccine intent in The Gambia
Source: Vaccine. 2018 Mar 20;36(13):1772–80. doi: 10.1016/j.vaccine.2018.02.037 (PMC5858151; doi:10.1016/j.vaccine.2018.02.037)
Supplement: Supplementary data 1 [file mmc1.docx]

**Appendix A: Acceptability of intranasal live attenuated influenza vaccine, influenza knowledge and vaccine intent in The Gambia – Questionnaire**

| **Question wording** | **Answer options** |
| --- | --- |
| Interview Site | 1, Sukuta \| 2, Faji Kunda |
| **Eligibility criteria** | 1, Yes \| 0, No |
| Is the interview being given in Mandinka? | 1, Yes \| 0, No |
| Is the participant female? | 1, Yes \| 0, No |
| Is the participant aged 18 or over? | 1, Yes \| 0, No |
| Does the participant have at least one child under the age of 5? | 1, Yes \| 0, No |
| Is the participant eligible to take part in the survey? | 1, Yes \| 0, No |
| **Attitudes to vaccination in pregnancy and childhood** |  |
| Were you ever asked to take part in the NASIMMUNE study? | 1, Yes \| 0, No |
| Do you have a child who took part in the NASIMMUNE study? | 1, Yes \| 0, No *(Yes = exposure group, No = control group)* |
| Were you ever asked to take part in the PROPEL study? | 1, Yes \| 0, No |
| Did you ever take part in the PROPEL study | 1, Yes \| 0, No |
| Have you ever been given a vaccine whilst pregnant? | 1, Yes \| 0, No \| 9, Don't know/can't recall |
| Have you ever been given a vaccine during pregnancy as part of a research study? | 1, Yes \| 0, No \| 9, Don't know/can't recall |
| Did your children get all their routine childhood vaccines? | 1, Received all \| 2, Received most \| 3, Received some \| 4, Received none \| 9, Cannot recall/unwilling to disclose |
| Did you know that there are vaccines that can protect people from the flu germ? | 1, Yes \| 0, No |
| Do you agree or disagree with this statement: "If I was pregnant, I would get a flu vaccine if it was free"? | 1, Agree Strongly \| 2, Agree \| 3, Disagree\| 4, Disagree Strongly \| 9, Don't know |
| If agreed with statement: Why would you get a flu vaccine in pregnancy?  *(open question without prompts)* | 1, Vaccines are effective \| 2, Flu is dangerous \| 3, Vaccines are safe \| 4, I trust healthcare professionals' advice \| 5, Vaccines are convenient and easy to get \| 6, I am not concerned about side-effects \| 7, Novelty/new technology \| 8, Other - specify \| 9, Don't know  *(unprompted answers categorised by interviewer into categories – multiple answers allowed)* |
| If disagreed with statement: Why would you decline a flu vaccine in pregnancy?  *(open question without prompts)* | 1, Vaccines do not work \| 2, Vaccines are not safe \| 3, I don't trust new vaccines \| 4, I would need to speak to my spouse/family first \| 5, Injections are painful \| 6, Vaccines are not important \| 7, Needles are not safe \| 8, The risk or side-effects is too great \| 9, Flu is not dangerous \| 10, I do not need it/I am not sick \| 11, Inconvenient/practicality \| 12, Don't know \| 13, Other – specify  *(unprompted answers categorised by interviewer into categories – multiple answers allowed)* |
| How many vaccines do you think it is safe to get in pregnancy? | 1, None \| 2, One \| 3, Two \| 4, Three or more \| 9, Don't know |
| Do you agree or disagree with this statement: "I would you get a flu vaccine for my child under 5, every year, if it was free"? | 1, Agree Strongly \| 2, Agree \| 3, Disagree\| 4, Disagree Strongly \| 9, Don't know |
| If agreed with statement: Why would you get a flu vaccine for your child?  *(open question without prompts)* | 1, Vaccines are effective \| 2, Flu is dangerous \| 3, Vaccines are safe \| 4, I trust healthcare professionals' advice \| 5, Vaccines are convenient and easy to get \| 6, I am not concerned about side-effects \| 7, Novelty/new technology \| 8, Other - specify \| 9, Don't know  *(unprompted answers categorised by interviewer into categories – multiple answers allowed)* |
| If disagreed with statement: Why would you decline a flu vaccine for your child?  *(open question without prompts)* | 1, Vaccines do not work \| 2, Vaccines are not safe \| 3, I do not trust new vaccines \| 4, I would need to speak to my spouse/family first \| 5, Injections are painful \| 6, Vaccines are not important \| 7, Needles are not safe \| 8, The risk or side-effects is too great \| 9, Flu is not dangerous \| 10, They do not need it/they are not sick \| 11, Inconvenient/practicality \| 12, Don't know \| 13, Other - specify  *(unprompted answers categorised by interviewer into categories – multiple answers allowed)* |
| **Influenza Knowledge (score out of 15 points)** |  |
| What are the symptoms of flu?  *(open question without prompts)* | 0, Didn't mention cough, sore throat or fever \| 1, Mentioned one of cough, sore throat and fever \| 2, Mentioned two of cough, sore throat and fever \| 3, Mentioned three of cough, sore throat and fever |
| Can flu be caught from another person? | 1, Yes \| 0, No |
| Can flu be caught from food? | 1, Yes \| 0, No |
| Can flu be caught from mosquitos? | 1, Yes \| 0, No |
| Which of these could cause flu? *(options provided)* | 1, Cold weather \| 2, Smoke in the house \| 3, Living in a crowded room or house |
| Can you prevent flu by taking herbs? | 1, Yes \| 0, No |
| Can you prevent flu by hand washing? | 1, Yes \| 0, No |
| Can you prevent others from catching flu by covering your mouth when coughing or sneezing? | 1, Yes \| 0, No |
| Which of these two people would flu be more dangerous for? *(options provided)* | 1, A pregnant woman \| 0, A 20 year old man |
| Which of these two people would flu be more dangerous for? *(options provided)* | 1, A 75 year old woman \| 0, A 50 year old woman |
| Which of these two people would flu be more dangerous for? *(options provided)* | 1, A 9 year old boy \| 0, A 6 month old baby |
| What is the cure for flu? *(options provided)* | 1, Medication \| 2, Herbs \| 3, Nothing/no treatment |
| How often do you need to get a flu vaccine in order to be protected? *(options provided)* | 1, Once only \| 2, Every year \| 3, Every five years |
| **Health Beliefs related to influenza and influenza vaccination** |  |
| "If I was pregnant but hadn't been given the flu vaccine, I would expect to get flu" | 1, Agree Strongly \| 2, Agree \| 3, Disagree\| 4, Disagree Strongly \| 9, Don't know |
| "If I refused to get the flu vaccine for my child under 5, but then they got sick with flu, I would be angry with myself" | 1, Agree Strongly \| 2, Agree \| 3, Disagree\| 4, Disagree Strongly \| 9, Don't know |
| "If I got a flu vaccine during pregnancy, the vaccine could give me flu" | 1, Agree Strongly \| 2, Agree \| 3, Disagree\| 4, Disagree Strongly \| 9, Don't know |
| "The flu vaccine is unsafe for children" | 1, Agree Strongly \| 2, Agree \| 3, Disagree\| 4, Disagree Strongly \| 9, Don't know |
| "If my child under 5 was given the flu vaccine, it would prevent them catching flu" | 1, Agree Strongly \| 2, Agree \| 3, Disagree\| 4, Disagree Strongly \| 9, Don't know |
| "If my child under 5 caught flu, it would be more mild than in the general public" | 1, Agree Strongly \| 2, Agree \| 3, Disagree\| 4, Disagree Strongly \| 9, Don't know |
| "If I was pregnant and refused to get the flu vaccine, but then got sick with flu, I would be angry with myself" | 1, Agree Strongly \| 2, Agree \| 3, Disagree\| 4, Disagree Strongly \| 9, Don't know |
| "If a nurse or doctor recommended the flu vaccine during pregnancy or for my child under 5, I would agree to get it because of what they said" | 1, Agree Strongly \| 2, Agree \| 3, Disagree\| 4, Disagree Strongly \| 9, Don't know |
| "The flu vaccine is unsafe during pregnancy" | 1, Agree Strongly \| 2, Agree \| 3, Disagree\| 4, Disagree Strongly \| 9, Don't know |
| "If I had been given a flu vaccine during pregnancy, it would prevent me catching flu" | 1, Agree Strongly \| 2, Agree \| 3, Disagree\| 4, Disagree Strongly \| 9, Don't know |
| "If my child under 5 hadn't been given the flu vaccine, I would worry about them getting flu?" | 1, Agree Strongly \| 2, Agree \| 3, Disagree\| 4, Disagree Strongly \| 9, Don't know |
| "If my child under 5 had been given the flu vaccine, the vaccine could give them flu" | 1, Agree Strongly \| 2, Agree \| 3, Disagree\| 4, Disagree Strongly \| 9, Don't know |
| "If my friends or relatives recommended the flu vaccine during pregnancy or for my child under 5, I would get it because of what they said" | 1, Agree Strongly \| 2, Agree \| 3, Disagree\| 4, Disagree Strongly \| 9, Don't know |
| "If my child under 5 had not been given the flu vaccine, I would expect them to get flu" | 1, Agree Strongly \| 2, Agree \| 3, Disagree\| 4, Disagree Strongly \| 9, Don't know |
| "If I was pregnant and hadn't been given the flu vaccine, I would worry about getting flu?" | 1, Agree Strongly \| 2, Agree \| 3, Disagree\| 4, Disagree Strongly \| 9, Don't know |
| "If I was pregnant and caught flu, it would be more mild than in the general public" | 1, Agree Strongly \| 2, Agree \| 3, Disagree\| 4, Disagree Strongly \| 9, Don't know |
| "If I got a flu vaccine during pregnancy, it would protect my baby from getting flu in the first few months of life" | 1, Agree Strongly \| 2, Agree \| 3, Disagree\| 4, Disagree Strongly \| 9, Don't know |
| "If my child under 5 caught flu, they might need to be admitted to hospital" | 1, Agree Strongly \| 2, Agree \| 3, Disagree\| 4, Disagree Strongly \| 9, Don't know |
| **Health seeking behaviour** |  |
| If your child has a FEVER, how would you decide what to do? *(prompting allowed)* | 1, Decide alone without discussing with anyone \| 2, Decide after discussion with husband \| 3, Decide after discussion with friends or neighbours \| 4, Decide after discussion with another family member(s) \| 5, Decide after discussion with the child's teacher/day carer \| 6, Decide after telephoning a clinic/hospital \| 7, Decide based on other information (specify) \| 9, Don't know |
| If your child has a COUGH, how would you decide what to do? *(prompting allowed)* | 1, Decide alone without discussing with anyone \| 2, Decide after discussion with husband \| 3, Decide after discussion with friends or neighbours \| 4, Decide after discussion with another family member(s) \| 5, Decide after discussion with the child's teacher/day carer \| 6, Decide after telephoning a clinic/hospital \| 7, Decide based on other information (specify) \| 9, Don't know |
| If your child has a SORE THROAT, how would you decide what to do? *(prompting allowed)* | 1, Decide alone without discussing with anyone \| 2, Decide after discussion with husband \| 3, Decide after discussion with friends or neighbours \| 4, Decide after discussion with another family member(s) \| 5, Decide after discussion with the child's teacher/day carer \| 6, Decide after telephoning a clinic/hospital \| 7, Decide based on other information (specify) \| 9, Don't know |
| If your child has a FEVER, what is the first place you will go for healthcare? *(prompting allowed)* | 1, Public health centre or hospital \| 2, Private health centre or hospital \| 3, Pharmacy \| 4, Other shop/vendor \| 5, Traditional healer \| 6, Other - specify \| 9, Don't know |
| If your child has a COUGH, what is the first place you will go for healthcare? *(prompting allowed)* | 1, Public health centre or hospital \| 2, Private health centre or hospital \| 3, Pharmacy \| 4, Other shop/vendor \| 5, Traditional healer \| 6, Other - specify \| 9, Don't know |
| If your child has a SORE THROAT, what is the first place you will go for healthcare? *(prompting allowed)* | 1, Public health centre or hospital \| 2, Private health centre or hospital \| 3, Pharmacy \| 4, Other shop/vendor \| 5, Traditional healer \| 6, Other - specify \| 9, Don't know |
| If your child had a FEVER, how long before you would go for healthcare? *(prompting allowed)* | 1, Same day \| 2, Next day \| 3, 2 days \| 4, 3-5 days \| 5, 5-7 days \| 6, Over a week \| 7, Wouldn't seek healthcare |
| If your child had a COUGH, how long before you would go for healthcare? *(prompting allowed)* | 1, Same day \| 2, Next day \| 3, 2 days \| 4, 3-5 days \| 5, 5-7 days \| 6, Over a week \| 7, Wouldn't seek healthcare |
| If your child had a SORE THROAT, how long before you would go for healthcare? *(prompting allowed)* | 1, Same day \| 2, Next day \| 3, 2 days \| 4, 3-5 days \| 5, 5-7 days \| 6, Over a week \| 7, Wouldn't seek healthcare |
| **Knowledge and attitudes towards intranasal LAIV (for participants whose children were not in NASIMMUNE)** |  |
| There is a vaccine against the flu germ that is delivered as a small spray into each nostril. Did you know that? | 1, Yes \| 0, No |
| If given a choice for your child between a flu vaccine injection and a nasal spray, which would you choose? | 1, Injection \| 2, Nasal spray \| 9, Don't know |
| If injection: Why would you prefer an injection for your child?  *(open question without prompts)* | 1, Injections are more effective \| 2, Don't know enough about it \| 3, Doesn't want a spray in nose of child \| 4, Nasal spray wouldn't work \| 5, More risks with nasal spray \| 6, More used to injections \| 7, Other - specify \| 9, Don't know  *(unprompted answers categorised by interviewer into categories – multiple answers allowed)* |
| If nasal spray: Why would you prefer a nasal spray for your child?  *(open question without prompts)* | 1, No needle required \| 2, Less painful \| 3, Easier to give \| 4, Fewer risks associated \| 5, More effective \| 6, Novelty/new technology \| 7, Other - specify \| 9, Don't know  *(unprompted answers categorised by interviewer into categories – multiple answers allowed)* |
| **Knowledge and attitudes towards intranasal LAIV (for participants whose children were in NASIMMUNE)** |  |
| Do you think the nasal spray is more distressing for your child than the injection? | 1, More distressing \| 2, Equally distressing \|3 , Less distressing \| 9, Don't know/can't remember |
| Do you think the nasal spray is safer than the injection? | 1, Safer \| 2, Equally safe \| 3, Less safe \| 9, Don't know/can't remember |
| Do you think the nasal spray is easier to give than the injection? | 1, Easier \| 2, Equally easy \| 3, Harder \| 9, Don't know/can't remember |
| If given a choice for your child between a flu vaccine injection and a nasal spray, which would you prefer? | 1, Injection \| 2, Nasal spray \| 9, Don't know |
| If injection: Why would you prefer an injection for your child?  *(open question without prompts)* | 1, Injections are more effective \| 2, Don't know enough about it \| 3, Doesn't want a spray in nose of child \| 4, Nasal spray wouldn't work \| 5, More risks with nasal spray \| 6, More used to injections \| 7, Other - specify \| 9, Don't know  *(unprompted answers categorised by interviewer into categories – multiple answers allowed)* |
| If nasal spray: Why would you prefer a nasal spray for your child?  *(open question without prompts)* | 1, No needle required \| 2, Less painful \| 3, Easier to give \| 4, Fewer risks associated \| 5, More effective \| 6, Novelty/new technology \| 7, Other - specify \| 9, Don't know  *(unprompted answers categorised by interviewer into categories – multiple answers allowed)* |
| **Demographics** |  |
| How old are you (in years)? | Numerical |
| How many children have you had in your life? | Numerical |
| Are you the first, second, third or fourth wife? | 0, Never married \| 1, First and only wife \| 2, First wife (not only) \| 3, Second wife \| 4, Third wife \| 5, Fourth wife \| 9, Unwilling to say |
| What is your tribe? | 1, Mandinka \| 2, Wolof \| 3, Fula \| 4, Jola \| 5, Serehule \| 6, Serere \| 7, Manjago \| 8, Other - specify \| 9, Don't know/unwilling to say |
| How many people sleep in your household (same roof, not compound)? | Numerical |
| Are you currently pregnant? | 1, Yes \| 0, No \| 9, Unsure/unwilling to say |
| Is anyone (not including you) in your compound currently pregnant? | 1, Yes \| 0, No \| 9, Unsure/unwilling to say |
| How many years of English school have you completed? | 1, None \| 2, Arabic school only \|3, Some primary school \| 4, Completed primary school \| 5, Some upper school \| 6, Completed upper school \| 7, Completed an undergraduate degree \| 8, Completed a post-graduate degree \| 9, Don't know/unwilling to say |
| What is your occupation? | 1, None \| 2, Student \| 3, Self-employed (market trader/vendor) \| 4, Self-employed (skilled trade/taxi driver/small business owner) \| 5, Daily wage earner (paid daily by an employer) \| 6, Salaried wage earner (paid monthly by an employer) \| 7 , Other (specify) \| 8, Don't know/unwilling to say |
| How many years of English school has your husband completed? | 1, None \| 2, Arabic school only \|3, Some primary school \| 4, Completed primary school \| 5, Some upper school \| 6, Completed upper school \| 7, Completed an undergraduate degree \| 8, Completed a post-graduate degree \| 9, Don't know/unwilling to say |
| What is your husband's occupation? | 1, None \| 2, Student \| 3, Self-employed (market trader/vendor) \| 4, Self-employed (skilled trade/taxi driver/small business owner) \| 5, Daily wage earner (paid daily by an employer) \| 6, Salaried wage earner (paid monthly by an employer) \| 7 , Other (specify) \| 8, Don't know/unwilling to say |
| What is your household (family) income per month? | 1, Less than D500 \| 2, D500-D999 \| 3, D1,000-D2,499 \| 4, D2,500-D4,999 \| 5, D5,000-D7,499 \| 6, D7,500-D9,999 \| 7, D10,000-D24,999 \| 8, Greater than D25,000 \| 9, Unable or unwilling to say |

**Table 1** Data collection tool used in face-to-face interviews – electronic data capture used on tablet computers using REDCap™.
